# HIV transcription persists in the brain of virally suppressed people with HIV

**DOI:** 10.1371/journal.ppat.1012446

**Published:** 2024-08-08

**Authors:** Janna Jamal Eddine, Thomas A. Angelovich, Jingling Zhou, Sarah J. Byrnes, Carolin Tumpach, Nadia Saraya, Emily Chalmers, Rory A. Shepherd, Abigail Tan, Stephanie Marinis, Paul R. Gorry, Jacob D. Estes, Bruce J. Brew, Sharon R. Lewin, Sushama Telwatte, Michael Roche, Melissa J. Churchill

**Affiliations:** 1 Infectious and Inflammatory Diseases, School of Health and Biomedical Sciences, RMIT University; Melbourne, Australia; 2 Life Sciences Discipline, Burnet Institute; Melbourne, Australia; 3 Department of Infectious Diseases, The University of Melbourne at the Peter Doherty Institute for Infection and Immunity, Melbourne, Australia; 4 Department of Microbiology and Immunology, The University of Melbourne at the Peter Doherty Institute for Infection and Immunity, Melbourne, Australia; 5 Vaccine & Gene Therapy Institute, Oregon Health & Science University, United States of America; 6 Peter Duncan Neurosciences Unit, Departments of Neurology and Immunology St Vincent’s Hospital, Sydney, University of New South Wales and University of Notre Dame; Sydney, Australia; 7 Department of Infectious Diseases, Alfred Hospital and Monash University; Melbourne, Australia; 8 Victorian Infectious Diseases Service, Royal Melbourne Hospital at the Peter Doherty Institute for Infection and Immunity, Melbourne, Australia; 9 Departments of Microbiology and Medicine, Monash University; Melbourne, Australia; NIH, NIAID, UNITED STATES OF AMERICA

## Abstract

HIV persistence in the brain is a barrier to cure, and potentially contributes to HIV-associated neurocognitive disorders. Whether HIV transcription persists in the brain despite viral suppression with antiretroviral therapy (ART) and is subject to the same blocks to transcription seen in other tissues and blood, is unclear. Here, we quantified the level of HIV transcripts in frontal cortex tissue from virally suppressed or non-virally suppressed people with HIV (PWH).

HIV transcriptional profiling of frontal cortex brain tissue (and PBMCs where available) from virally suppressed (n = 11) and non-virally suppressed PWH (n = 13) was performed using digital polymerase chain reaction assays (dPCR). CD68+ myeloid cells or CD3+ T cells expressing HIV p24 protein present in frontal cortex tissue was detected using multiplex immunofluorescence imaging.

Frontal cortex brain tissue from PWH had HIV TAR (n = 23/24) and Long-LTR (n = 20/24) transcripts. Completion of HIV transcription was evident in brain tissue from 12/13 non-virally suppressed PWH and from 5/11 virally suppressed PWH, with HIV p24+CD68+ cells detected in these individuals. While a block to proximal elongation was present in frontal cortex tissue from both PWH groups, this block was more extensive in virally suppressed PWH.

These findings suggest that the brain is a transcriptionally active HIV reservoir in a subset of virally suppressed PWH.

## Author summary

HIV reservoirs have been shown to persist within the brain of people with HIV (PWH) despite antiretroviral therapy (ART). Whether this reservoir is transcriptionally or translationally active within the brain during ART remains unclear. We demonstrated that transcription persists in brain of virally suppressed PWH, although at a lower level than reservoirs in circulating CD4+ T cells. Furthermore, while HIV transcripts related to initiation of HIV transcription were detected in brain tissue from all virally suppressed PWH assessed, transcripts related to the completion of transcription was only evident in a small subset. Within this subset, we further demonstrated the presence of HIV proteins within resident brain cells. Our study characterises the persistence of ongoing HIV transcription and translation within the brain of PWH despite viral suppression. This may provide a source of neuroinflammation and contribute to the development of HIV associated neurocognitive disorders.

## Introduction

A major barrier to HIV cure is the presence of reservoirs of integrated HIV in tissues. These persistent reservoirs contribute to comorbid disease and are not abrogated by viral suppression with antiretroviral therapy (ART). Tissue reservoirs, including the brain, serve as important sites of HIV persistence which are often more difficult to target with ART compared to peripheral blood, and contain unique microenvironments that may drive localised tissue pathology and disease [1]. Importantly, 20–30% of people with HIV (PWH) develop HIV-associated neurocognitive disorders (HAND) despite viral suppression with ART, suggesting the presence of active viral reservoirs in the brain [2]. Understanding the nature of viral persistence, and the potential for ongoing HIV transcription and virus production in the brain, is essential for elucidating the role of the central nervous system (CNS) as a reservoir of replication competent HIV and in the pathogenesis of HAND.

HIV invades the CNS during acute infection, likely via the transmigration of infected circulating CD4+ T cells and possibly monocytes across the blood-brain barrier, facilitating the infection of parenchymal microglia, astrocytes and pericytes [3–7]. Recent studies have demonstrated that both intact and defective HIV proviral DNA persists in the brain of virally suppressed PWH [8–11], supporting the presence of a potentially replication competent viral reservoir. Furthermore, a study utilizing autopsy brain tissue identified replication competent viral genomes *ex vivo* using a quantitative viral outgrowth assay, following isolation of myeloid cells from the brain [12]. While this study demonstrated the presence of intact HIV genomes in the brain, whether these genomes are transcriptionally active *in vivo* remains unclear.

Blocks to HIV transcription in latently infected CD4+ T cells from blood and lymphoid tissues have been identified [13,14]. These blocks occur at the transcriptional elongation, completion (polyadenylation) and splicing stages, with polyadenylation and splicing required to produce infectious virions. Blocks to HIV transcription differ between cellular and tissue compartments, with CD4+ T cells in gut tissue exhibiting different blocks to transcription than those in circulating CD4+ T cells [15]. The nature of the HIV transcriptional landscape in the CNS of PWH is unclear.

Here we utilised an established digital PCR based approach [14,16] to characterise the transcriptional profile of HIV genomes in the brain of virally suppressed and non-virally suppressed PWH.

## Results

### Proximal and distal blocks to HIV RNA transcription are present in brain tissue of non-virally suppressed and virally suppressed PWH

To characterise the HIV transcriptional landscape in the brain of PWH, RNA transcripts (HIV TAR, Long-LTR, Pol, PolyA and Tat/Rev) were quantified by the nanowell digital PCR-based HIV transcript assays using brain tissue from PWH [16]. Fresh frozen autopsy frontal cortex brain tissue from viremic (non-virally suppressed; n = 13; median plasma viral load: 61,223 [8,557–558,810] HIV RNA copies/mL) and virally suppressed PWH (n = 11; all undetectable plasma viral load <50 HIV RNA copies/mL) was provided by the National NeuroAIDS Tissue Consortium (NNTC; Table 1). Viral suppression was defined as >2 year of sustained ART treatment with undetectable plasma viral loads (1 blip < 250 copies HIV RNA/mL at > 6 months from autopsy allowed). The median time of viral suppression was 3.93 years. One non-virally suppressed PWH (nVS PWH 13) had plasma viral loads <100 HIV RNA copies/mL and several nVS PWH were treated with ART at death. However, these individuals did not have an undetectable viral load for >2 years prior to death and therefore were not included in the virally suppressed PWH group. No participants had systemic infection at the time of death.

**Table 1:** Clinical characteristics.

PWH	Age	Sex	Viral load	Viral suppression (years)^b^	CD4^+^ T cells	Nadir CD4^+^ T cells	ARV (at autopsy)	CPE score^f^
**Virally suppressed**								
VS PWH 1 ^c^	67	M	UD	7.32	355	30	ATV, EPZ, RTV	6
VS PWH 2 ^c^	64	M	UD	3.40	1043	60	EFV, TRU	7
VS PWH 3 ^d^	56	F	UD	6.21	239	90	3TC, ABC, NVP	9
VS PWH 4 ^c^	46	M	UD	3.76	61	61	DRV, RGV, RTV, TRU	11
VS PWH 5 ^c^	59	M	UD	2.47	328	161	RGV, TRU	7
VS PWH 6 ^c^	58	M	UD	2.57	213	a	CBV, NFV	5
VS PWH 7	62	M	UD	13.63	274	65	3TC, ABC, RGV	8
VS PWH 8	52	M	UD	6.04	417	78	EFV, KTA, TZV, ATR	7^g^
VS PWH 9	63	M	UD	9.15	140	54	3TC, EFV, TFV	6
VS PWH 10	62	M	UD	3.93	172	34	DTG, TRU	8
VS PWH 11	52	M	UD	2.58	383	230	3TC, D4T, EFV	7
**Median**	**59 (52–63)**		**UD**	**3.93 (2.58–7.32)**	**274 (172–383)**	**63 (49–108)**		
**Non-virally suppressed**								
nVS PWH 1	46	M	750,000	Never	6	6	None	N/A
nVS PWH 2	42	M	25,022	Never	17	4	None	N/A
nVS PWH 3	42	M	688	Never	441	105	EPZ, PRE	5^g^
nVS PWH 4	55	F	17,387	Never	8	a	FTC, RGV, TFV, TMC	7^g^
nVS PWH 5	56	M	61,223	Never	24	8	3TC, ABC, T20	5^g^
nVS PWH 6	49	M	750,000	Never	3	a	D4T, KTA	6
nVS PWH 7	48	M	750,000	Never	2	2	None	N/A
nVS PWH 8	47	F	367,620	Never	2	1	None	N/A
nVS PWH 9	62	M	222,840	Never	8	8	3TC, ABC, D4T	7
nVS PWH 10	39	M	72,125	Never	1	0	3TC, D4T, DDI	6
nVS PWH 11	44	F	14,286	Never	290	a	None	N/A
nVS PWH 12	35	M	2,827	Never	211	a	3TC, D4T, IDV	7
nVS PWH 13	43	M	64^e^	Never	110	31	DRV, RTV, TRU	8
**Median**	**46 (42–52)**		**61,223 (8,557–558,810)**	**-**	**8.00 (2.50–161)**	**8.00 (1.50–68.0)**		

**3TC: lamivudine, ABC: abacavir; ARV: antiretroviral; ATR: atripla; ATV: atazanavir; CBV: combivir; CPE: CNS penetration effectiveness score; d4T: stavudine; DDI: didanosine; DRV: darunavir; DTG: dolutegravir; EFV: efavirenz; EPZ: epzicom; FTC: emtricitabine; IDV: indinavir; KTA: biktarvy; N/A; not applicable; NFV: nelfinavir; NVP: nevirapine; nVS: non-virally suppressed; PWH: people with HIV; RGV: raltegravir; RTV: ritonavir; TFV: tenofovir; TMC: TMC-310911; TRU: truvada; T20: enfuvirtide; UD: undetectable (<50 HIV RNA copies/mL); VS: virally suppressed**

^**a**^ **missing data,**

^**b**^ **Viral suppression defined by > 2 years undetectable viral load,**

^**c**^ **PBMCs tested,**

^**d**^ **Excluded from HIV transcript analysis,**

^**e**^ **not undetectable viral load for > 2 years,**

^**f**^ **CPE score ≤5 (low penetration), 6–8 (medium penetration) and ≥9 (high penetration) calculated as per [17],**

^**g**^ **CPE score for one or more ARV not defined; medians and interquartile ranges shown.**

**Fig 1:**
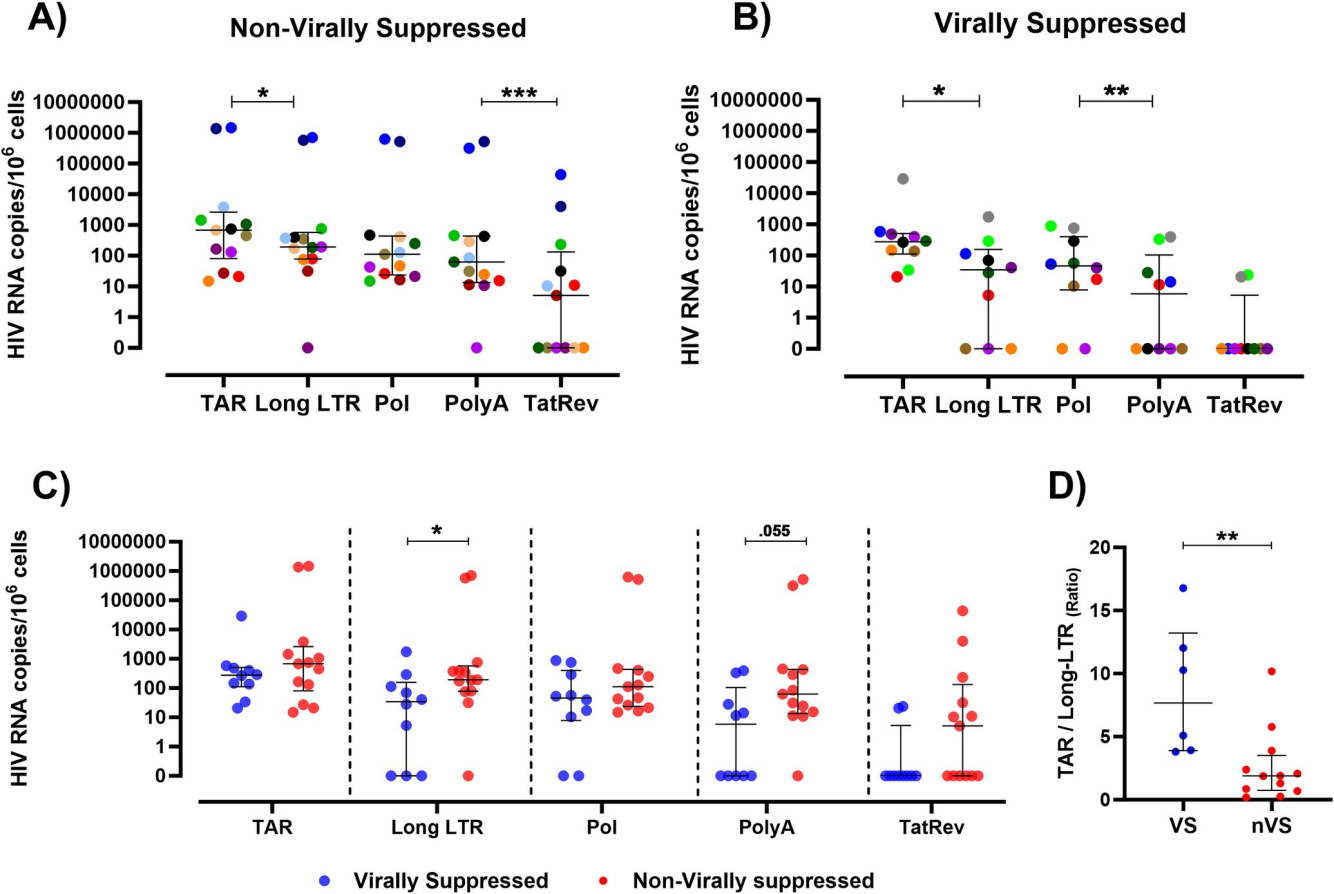
HIV RNA transcripts are present in brain tissue from non-virally suppressed and virally suppressed PWH. HIV RNA transcripts for HIV TAR, Long-LTR, Pol, PolyA and Tat/Rev quantified by QIAcuity digital PCR from frontal cortex brain tissue from **(A)** non-virally suppressed (n = 13) or **(B)** virally suppressed PWH (n = 10). HIV transcripts standardised to 10^6^ cell equivalents as measured by RRP30 DNA. Comparisons made using non-parametric paired Wilcoxon-tests where colored symbols represent individual PWH. **(C)** Comparative analysis of TAR, Long-LTR, Pol, PolyA and multiply spliced Tat/Rev HIV transcripts between virally suppressed (blue) and non-virally suppressed PWH (red). **(D)** Comparative analysis of HIV TAR RNA/Long-LTR RNA ratio in frontal cortex tissue from virally suppressed (VS; n = 6) or non-virally suppressed PWH (nVS; n = 11). Comparisons made using non-parametric unpaired Mann-Whitney tests. Median and interquartile ranges shown. *P<0.05, **P<0.01, ***P<0.001.

We performed HIV transcriptional profiling assays on RNA isolated from the frontal cortex brain tissue utilising a digital PCR approach that utilises successive primer/probe sets to identify transcriptional initiation (TAR), proximal elongation (R-U5-pre-Gag; “Long-LTR”), elongation (Pol), transcriptional completion (PolyA), and multiple splicing (Tat/Rev). The level of each transcript was normalised to 10^6^ cell equivalents by RPP30 DNA (Fig 1), μg RNA and DNA mass in matched samples, as previously described [14,16] (S1 Fig).

The majority of non-virally suppressed PWH had detectable TAR, Long-LTR, Pol and Poly A HIV transcripts in the frontal cortex of the brain (Fig 1A). Multiply spliced transcripts (Tat/Rev) were detected in a subset of non-virally suppressed PWH (7/13). Median levels of Long-LTR were 3.5-fold lower relative to TAR (medians: 192 vs 681 copies/10^6^ cell equivalents, P<0.001), suggesting a block to proximal elongation. Median levels of Tat/Rev were lower than PolyA (medians: 0.00 vs 62.7 copies/10^6^, P<0.01), indicating a block to multiple splicing. Levels of HIV Long-LTR, Pol and PolyA were similar in non-virally suppressed PWH.

HIV TAR transcripts were also detected in 10/11 brain tissue of virally suppressed PWH (Fig 1B). The virally suppressed PWH with no detectable TAR was excluded from further analysis as they also had no detectable levels of Long-LTR, but detectable HIV Pol RNA, potentially reflecting sequence polymorphisms in these regions. A proximal block to transcription was observed with levels of HIV Long-LTR transcripts ~8 fold lower than levels of HIV TAR in virally suppressed PWH (median: 34.4 vs 277 copies/10^6^ cells; P<0.05), suggesting inefficient proximal elongation. Levels of HIV Long-LTR were similar to levels of Pol (median: 34.4 vs 46.4 copies/10^6^ cells, P>0.05). Levels of HIV PolyA were significantly lower than Pol in brain tissue from virally suppressed PWH (5.8 vs 46.4 copies/10^6^ cells; P<0.01), demonstrating a block to transcription completion. Notably, HIV Tat/Rev transcripts were detected in brain tissue from 2/10 virally suppressed PWH, indicating that the generation of multiply spliced transcripts occurs in the frontal cortex of a subset of virally suppressed PWH. Interestingly, levels of HIV RNA transcripts in the brain of non-virally suppressed and virally suppressed PWH were similar, except for levels of Long-LTR (median nVS vs VS PWH: 192 vs 34.4 copies/10^6^ cells; P<0.05) and PolyA (median nVS vs VS PWH: 62.7 vs 5.8 copies/10^6^ cells; P = 0.055) which were higher in non-virally suppressed PWH (Fig 1C). The block between HIV TAR and Long-LTR was 5-fold higher in virally suppressed PWH than non-virally suppressed PWH, indicating a larger block to proximal elongation in PWH suppressed with ART (Fig 1D). Together, these data provide evidence that HIV transcription initiation, elongation, and completion, at least in some PWH, occurs in both non-virally suppressed and virally suppressed PWH regardless of long-term viral suppression with ART.

The total level of each HIV RNA transcript in the brain did not correlate with plasma levels of HIV RNA, CD4+ T cells in the blood or time between last plasma viral load test and death (S1 Table). No clear association between ART regimen or associated CNS penetrance effectiveness (CPE) scores at death and levels of HIV RNA transcripts were also observed (S1 Table) with all virally suppressed PWH with detectable Tat/Rev transcripts being treated with medium (CPE 6–8) or high (CPE >9) CPE ARVs at death.

**Fig 2:**
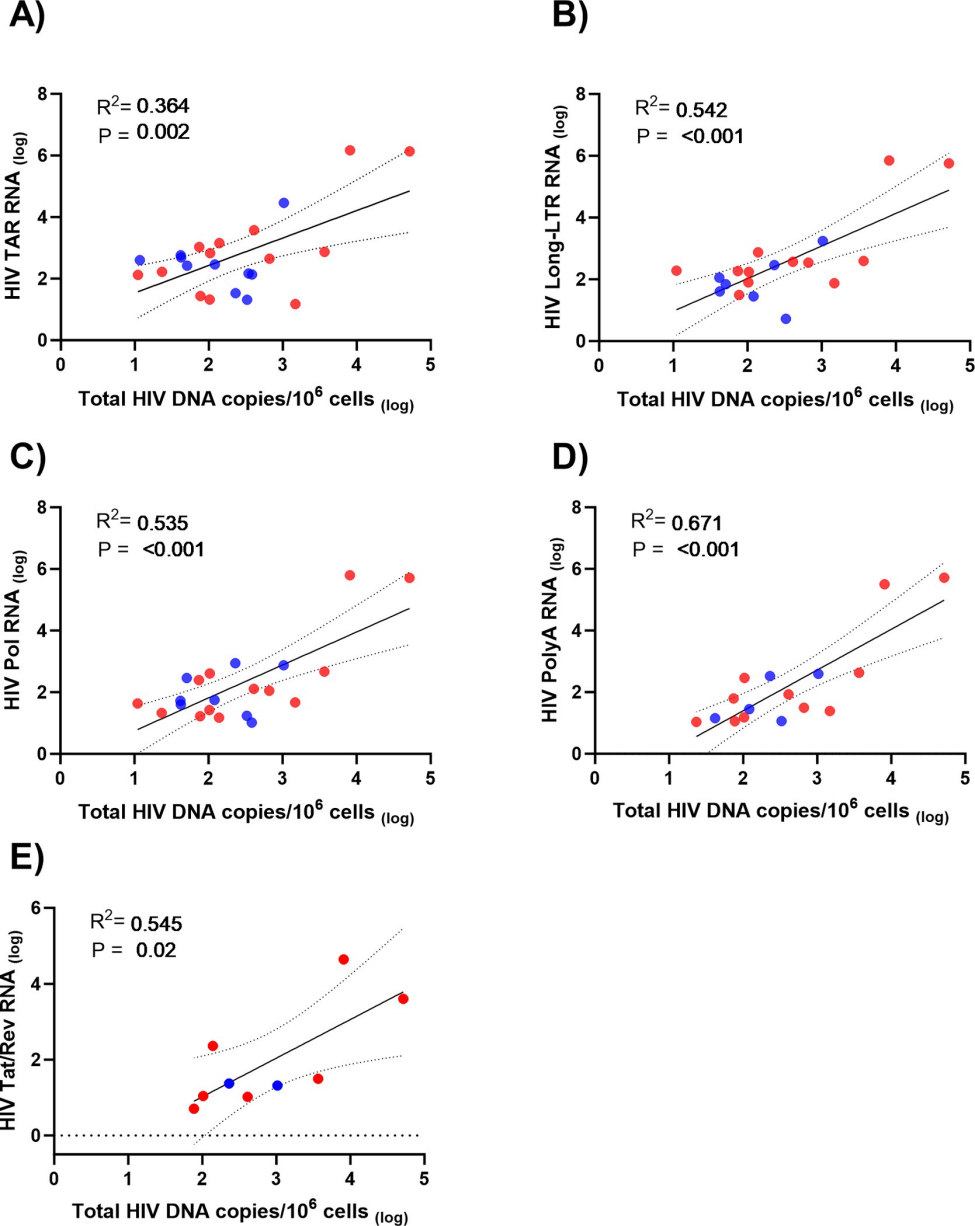
Levels of HIV RNA transcripts in brain tissue are directly associated with HIV total DNA levels in PWH. Linear regression analysis of HIV RNA transcription **(A)** initiation (TAR), **(B)** elongation (Long-LTR), **(C)** Pol, **(D)** completion (PolyA) and **(E)** multiply spliced Tat/Rev transcripts vs total HIV DNA levels in frontal cortex brain tissue as measured by digital PCR for virally suppressed (n = 2–14; blue symbols) and non-virally suppressed PWH (n = 7–13; red symbols). All parameters log transformed; R^2^, P values and 95% confidence intervals shown.

Notably, the total levels of each HIV RNA transcript was associated with total HIV DNA levels in the brain (P<0.05 for all; Fig 2), demonstrating that the level of HIV transcription is associated with reservoir size within the brain. HIV RNA transcripts were also associated with levels of intact proviral DNA (S2 Fig). Together, these data suggest that transcriptional activity is directly associated with HIV reservoir size in the brain.

### HIV viral p24 is present in brain tissue from virally suppressed PWH with completed HIV transcription

**Fig 3:**
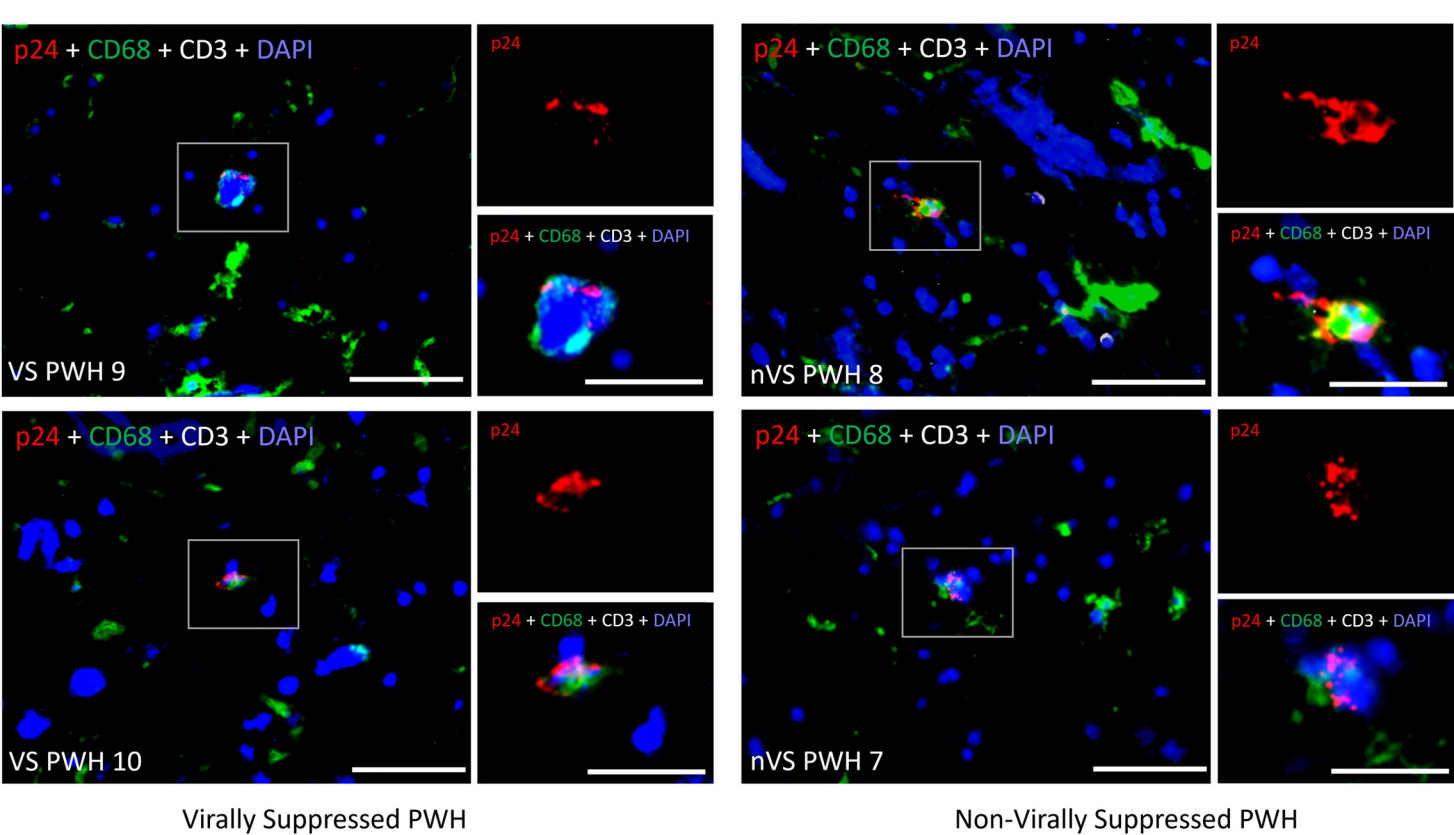
Expression of HIV p24 protein in human brain tissue from non-virally suppressed or virally suppressed PWH. Representative images of cell nuclei (blue), CD68+ myeloid cells (green), CD3+ T cells (white) and HIV p24 protein (red) labelled cells by immunofluorescence imaging from human frontal cortex brain tissue from virally suppressed PWH (VS PWH; left panels) and non-virally suppressed (nVS PWH; right panels; n = 2 per group shown). Donor ID shown in white text. Larger images (40× magnification; 50 μm scale bar) and insets (40× magnification + 3× digital zoom; 10 μm scale bar).

To confirm whether the presence of completed HIV transcription (as defined by the presence of PolyA and/or multiply spliced Tat/Rev HIV transcripts) is associated with translation of viral proteins in brain tissue, the presence of the p24 HIV capsid protein was measured by immunohistochemistry in matched formalin-fixed, paraffin-embedded (FFPE) brain tissue. Tissue was also co-labelled with CD68 and CD3 to identify myeloid cells and T cells, respectively. No matched FFPE tissue was available for non-virally suppressed PWH 1 and 13. HIV p24+ cells were detected in all non-virally suppressed and virally suppressed PWH with detectable HIV PolyA and/or Tat/Rev transcripts (representative images shown Fig 3). Subsequent co-localisation analysis demonstrated that parenchymal p24+ cells were CD68 expressing myeloid cells. A rare population of CD3+p24+ T cells were also detected in some individuals; however, these were restricted to vessels, likely reflecting infected peripheral T cells attached to vessel walls. Our results demonstrate that active (and in some cases completed) HIV transcription persists in the brain of PWH which has the potential to lead to viral protein production.

### HIV RNA transcripts in the frontal cortex of virally suppressed PWH are present at lower levels than those in PBMCs

**Fig 4:**
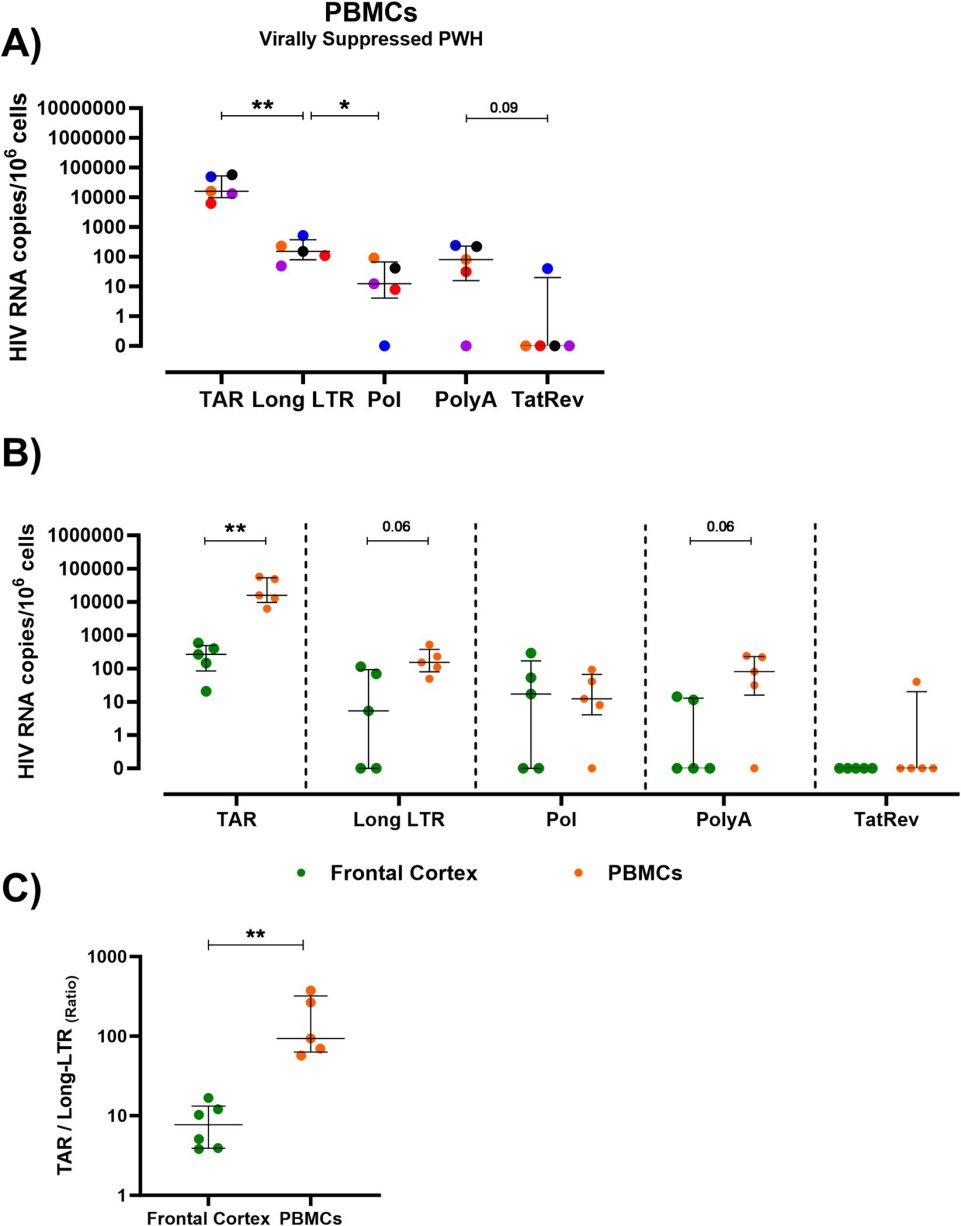
Comparative analysis of HIV RNA transcripts in PBMCs relative to frontal cortex brain tissue from virally suppressed PWH. **(A)** HIV RNA transcripts for HIV TAR, Long-LTR, Pol, PolyA and Tat/Rev quantified by QIAcuity digital PCR in PBMCs from virally suppressed PWH (n = 5). HIV transcripts standardised to 10^6^ cell equivalents as measured by RRP30 DNA. Colored symbols represent individual PWH. **(B)** Comparative analysis of levels of HIV RNA TAR, Long-LTR, Pol, PolyA and multiply spliced Tat/Rev transcripts between frontal cortex brain tissue (green) and PBMCs (orange) from virally suppressed PWH. **(C)** Comparison of ratio of TAR/LongLTR transcripts in frontal cortex and PBMCs from virally suppressed PWH (n = 5–6). Comparisons made using Mann-Whitney U tests. Median and interquartile ranges shown. *P<0.05, **P<0.01. P<0.05 considered statistically significant.

To compare how the levels and pattern of expression of HIV RNA transcripts in frontal cortex tissue relate to other compartments in the body, transcriptional profiling of matched PBMCs of virally suppressed PWH was performed. Virally suppressed PWH with matched PBMCs available were identified (n = 5). PBMCs from the virally suppressed PWH tested had detectable levels of HIV TAR (5/5), Long-LTR (5/5), Pol (4/5) and PolyA transcripts (4/5) with one individual harbouring multiply spliced Tat/Rev transcripts (1/5; Fig 4A). HIV Pol transcripts were not detected in PBMCs for one individual despite >700 ng of material screened (VS PWH 1: blue). As all other transcripts were present at levels ≥40 copies/10^6^ cells, this may reflect sequence polymorphisms for this individual in the Pol region. Similar to previous findings [14,15], PBMCs contained a similar pattern in levels of HIV RNA transcripts demonstrated by higher levels of TAR, followed by Long-LTR, Pol and PolyA with only one individual harbouring Tat/Rev transcripts (Fig 4A).

When comparing between compartments, PBMCs contained higher levels of TAR (median PBMCs vs frontal cortex: 15,902 vs 265.1 copies/10^6^ cells; P<0.01) and Long-LTR (median PBMCs vs frontal cortex: 152.3 vs 5.3 copies/10^6^ cells; P = 0.06) and PolyA (median PBMCs vs frontal cortex: 80.6 vs 0.0 copies/10^6^ cells; P = 0.06) transcripts than those in matched frontal cortex brain tissue (Fig 4B). The ratio of HIV TAR RNA relative to total HIV DNA was 10-fold higher in PBMCs than those present in the frontal cortex (S3 Fig; P<0.01). Similar to findings in frontal cortex tissue, a block to proximal elongation was also detected in PBMCs. However, the magnitude of the block was ~10-fold higher in PBMCs relative to frontal cortex tissue (Fig 4C). Together, these findings demonstrate that a block to proximal elongation exists in both PBMCs and frontal cortex tissue from virally suppressed PWH. However, the levels of HIV transcripts, and magnitude of the block to proximal elongation is larger in PBMCs relative to frontal cortex of the brain.

## Discussion

The role of the brain as an active reservoir of HIV in virally suppressed PWH is unclear. While intact proviral genomes are potentially capable of producing viral proteins [8,10,11], and the isolation of replication competent HIV have been demonstrated in brain tissue [12], whether these genomes are capable of producing virus *in vivo* confirming the brain as a tissue reservoir of HIV has not been established. Peripheral blood and non-CNS tissue sites harbour transcriptionally active reservoirs despite long-term viral suppression with ART which likely contributes to immune activation and inflammatory disease. Given that PWH continue to develop HAND, determining whether disease pathogenesis is driven by ongoing transcription in the brain warrants investigation.

In this study, we characterized the persistent HIV reservoir in the frontal cortex of the brain of both non-virally suppressed and virally suppressed PWH and demonstrate that active HIV transcription occurs. HIV RNA transcripts were detected in frontal cortex tissue of PWH regardless of viral suppression with ART, which mimics our findings in matched PBMCs and those of others in circulating CD4+ T cells and tissue reservoirs including the gut of virally suppressed PWH [14,15,18]. However, levels of HIV TAR transcripts in frontal cortex were largely similar between virally suppressed and non-virally suppressed PWH, demonstrating that long-term viral suppression with ART does not appear to be impacting the levels of HIV transcriptional initiation in the brain. Although blocks to HIV transcription were similar between virally suppressed and non-virally suppressed PWH, a block to transcriptional completion (i.e. Pol➔PolyA) was observed in virally suppressed PWH which was not present in non-virally suppressed individuals. Importantly, all HIV transcript species were detected in 2/11 virally suppressed and 7/13 non-virally suppressed PWH, demonstrating the presence of transcriptional completion and splicing in the brain regardless of viral suppression. Levels of transcripts in the brain were generally lower than those in matched PBMCs.

The presence of completion of transcription, splicing and HIV protein production suggests that the brain reservoir of HIV in virally suppressed PWH is not silent, but active in some individuals. Furthermore, this completion of transcription and protein production was associated with intact proviruses in the brain. We and others have demonstrated the presence of intact, potentially replication competent HIV proviruses in the brain of virally suppressed PWH despite long-term viral suppression with ART [8,10–12]. Recently, microglia isolated and enriched from the brains of PWH who had undergone rapid autopsy, were demonstrated to contain replication competent virus ex vivo in a viral outgrowth assay [12]. Whether these viruses can truly replicate in the brain *in vivo* and reseed peripheral cells and tissue is unknown and requires further investigation.

TAR transcripts were detected in the frontal cortex (and PBMCs of those tested) in 23/24 PWH regardless of viral suppression with ART. Immunofluorescence imaging demonstrated the presence of p24 predominantly in CD68+ myeloid cells, supporting our previously published findings that the major HIV reservoir in the frontal cortex of the brain is present in microglia and macrophages and not CD3+ T cells [8]. Microglia play a key role in sensing and responding pathogenic antigens in the brain. Therefore, sensing of HIV RNA by microglia/macrophages in the brain may induce cellular activation and contribute to neuroinflammation and neuropathology in PWH independent of HIV protein production or viral rebound. Double stranded (ds) HIV RNA is capable of inducing innate immune activation via viral sensing pathways including TLR-3, TLR-7, RIG-I and Mitochondrial antiviral-signaling protein (MAVS) pattern recognition receptors, resulting in induction of antiviral interferon signalling and proinflammatory cytokine production [19–21]. Specifically, HIV RNA has been shown to activate macrophages *ex vivo* in an IFN-I dependent manner via MAVS signalling, resulting in the production of proinflammatory cytokines [19]. Intron containing HIV RNA also induces immune activation in microglia cell lines independent of the generation of multiply spliced HIV RNA [22]. Cell associated HIV RNA in cerebral spinal fluid (CSF) is also associated with brain injury as measured by ^1^H magnetic resonance spectroscopy in frontal white matter [23], supporting a mechanism by which ongoing HIV transcription contributes to neuropathology and HAND.

The presence of RNA in the brain of a subset of virally suppressed PWH supports a recent study where levels of cell associated HIV gag RNA, as measured by ddPCR, were similar to those observed in this study (and at a similar ratio relative to integrated HIV DNA) in microglia isolated from the CNS of a PWH, as part of the rapid autopsy ‘last gift cohort’ [12]. While no transcriptional profiling was carried out, the data support our observations of ongoing transcription and the CNS as a transcriptionally active reservoir. The sources of HIV transcripts in brain tissue require further investigation, however, as our study utilised material from virally suppressed PWH that were suppressed for >2 years, viral persistence and transcription observed likely reflects ongoing activation that are not related to historical viruses. Our detection of p24+ cells also supports the potential of a translationally competent reservoir as the half-life of p24 is suggested to be approximately 30 days in the plasma and likely months, not years in tissue [24]. As this study utilised human autopsy material, our findings represent a single timepoint. It is possible (and likely) that the level of viral transcription does change over time. In individuals where HIV transcripts were not detected, they may have been present during earlier time points, or in other regions of the brain than those measured. However, the temporal expression of HIV transcripts cannot be sampled using autopsy samples and studies using well characterized non-human primate models may offer additional insight into changes in levels of viral transcripts in the brain over time.

It is possible that the transcripts detected in this study may have been generated from replication incompetent defective proviruses. Defective HIV proviruses have been shown to be capable of viral transcription and in some cases produce viral proteins following translation [25,26]. Specifically, HIV RNA transcripts produced in PBMCs have been previously associated with IL-6 and TNF-α production. Therefore, viral transcripts may be a contributor to ongoing immune activation in the brain of PWH, without the requirement for full transcription and/or translation processes. Defining the contribution of defective proviruses in the brain in the generation of HIV transcripts and potential neuropathology requires further investigation in larger dedicated studies.

Major proximal and distal blocks to transcription in the brain were similar to findings reported in CD4+ T cell reservoirs in blood and gut tissue [14,15,18]. We also identified that HIV transcripts in the brain of individuals were present at lower levels to those in matched PBMCs. These data could suggest that factors that maintain latency in the brain are similar to those in the periphery, including a lack of host elongation factors, splicing factors, aberrant nuclear export and/or impaired Tat activity [27–30]. Indeed, we observed that multiply spliced Tat/Rev transcript levels were lower than polyadenylated transcripts, suggesting a specific block to multiple splicing. This predicted lack of Tat may also explain the block in transcript elongation. It is unlikely that RNA decay will impact the presence/absence of transcripts being detected. The decay of all transcripts we assessed has been previously measured in CD4+ T cells of virally suppressed PWH [15]. The half-life for the majority of transcripts were similar. Tat-Rev is the most stable transcript, however, represents the smallest population of transcripts identified. Similarly, the presence of a polyA tail would increase the stability of polyadenylated transcripts [31], however these transcripts also represent a small fraction of the total transcripts quantified. Thus, differences in RNA stability alone do not account for the trends seen within the transcriptional profiling assay.

Our findings have significant clinical implications regarding both HIV cure and long-term treatment approaches. Levels of HIV transcripts in frontal cortex tissue did not correlate with the length of viral suppression. Moreover, no correlation or discernible pattern with CPE scores and detection of PolyA and Tat/Rev transcripts was observed, indicating that the detection of multiply spliced transcripts in frontal cortex tissue of virally suppressed PWH was not related to treatment with ARVs with low CNS penetrance at death. However, it is possible that transcription may be occurring within tissue niches with low concentrations of ART (as observed in peripheral tissues [32]) including frontal white matter, which is a major pathophysiological site of HAND and a reservoir of intact and defective HIV provirus in the brain [10]. Additional studies using spatial analysis of HIV RNA transcripts within tissue sections may resolve the specific contributions of local viral transcription in the brain on the surrounding cellular environment and potential cognitive dysfunction.

Virally suppressed PWH in this study had a relatively low median CD4 count of 274 cells/mm^3^. Therefore, it would be of interest to assess the level of viral transcription in individuals with circulating CD4 T+ cell counts >500 cells/mm^3^. Additionally, future studies understanding the impact of persistent low level viraemia present in individuals with non-suppressible viremia on HIV transcripts in brain tissue may inform on activity and contribution of viral reservoirs in the brain of PWH on neurocognitive disorders.

Our results also provide renewed support for novel treatment approaches to target ongoing HIV transcription which is largely not affected by current HIV treatment approaches. Therefore, selection of neuroART regimens with deeper CNS penetrance coupled with the generation of therapeutic strategies targeting HIV transcription may be required to truly silence ongoing HIV transcription in the brain. These data also have implications for latency reversal approaches in the CNS [33], and new latency reversing agents (LRAs) would have to overcome similar blocks as those seen in peripheral and lymphoid CD4+ T cells and penetrance issues of drugs into the brain [14,34].

Together, in this study we demonstrate the presence of a transcriptionally active HIV reservoir in the brain of a subset of virally suppressed PWH which may contribute, at least in part, to ongoing immune activation.

## Materials and methods

### Ethics statement

Fresh frozen and FFPE autopsy frontal cortex brain tissue and matched PBMCs (if available) was obtained with ethics approval and written approval from the National NeuroAIDS Tissue Consortium, USA (https://nntc.org) [35]. Tissues were processed under ethics approval (Royal Melbourne Institute of Technology (RMIT) University Human Research Ethics Committee #20843).

### Cohort

Fresh frozen and FFPE autopsy brain tissue from virally suppressed (n = 11) and non-virally suppressed (n = 13) PWH were utilised. The time between death and autopsy (post-mortem interval) was a median (IQR) 7.5 hours (5.0–18). CNS penetrance effectiveness was calculated by adding the relative penetrance scores of each ARV prescribed at time of death, as previously described [17].

### Genomic DNA/RNA extraction and reverse transcription

gDNA/RNA from fresh frozen frontal lobe brain tissue (~10 mg pieces) and matched PBMCs were extracted using the AllPrep DNA/RNA/miRNA universal kit for transcriptional profiling analysis (QIAGEN, Hilden, Germany) or DNA extraction kit (Agilent; Santa Clara, CA) for intact proviral DNA assay (IPDA) analysis. RNA and DNA concentrations and quality were measured using the Nanodrop 1000 spectrophotometer (Thermo Fisher Scientific, Waltham, MA). Isolated gDNA was used for IPDA and RPP30 quantification, as previously described [8]. Isolated RNA was reverse transcribed and used for quantification of HIV TAR, R-U5/pre-Gag (“Long LTR’), Pol, U3-R-polyA (“PolyA”) and multiply spliced Tat/Rev HIV transcripts, as previously described [14,15]. For efficient reverse transcription of short, prematurely terminated HIV transcripts limited to the TAR loop, reverse transcription from a linker molecule is necessary [36]. Therefore, a polyadenylation step was utilised prior to reverse transcription and dPCR for the TAR assay, as previously described [16].

### Quantification of HIV RNA transcripts in human brain tissue and PBMCs

Qiagen’s QIAcuity Four 5-plex digital PCR System (Qiagen) was utilised to detect HIV TAR, Long LTR, Pol, PolyA and Tat/Rev transcripts, as previously described [16]. Up to 1.5μg of RNA was screened for TAR and up to 2μg of RNA was screened for each remaining transcript. In instances where initial RNA input was low, additional gDNA and RNA was extracted, and results were averaged. Cycling conditions: 2mins at 95°C followed by 45 cycles of 95°C for 15°sec and 59°C for 30sec. Partitions with a FAM target were imaged with an exposure time of 400ms and partitions with a VIC target were imaged with an exposure time of 300ms. Gain was set to 6 for both channels. A six-assay synthetic HIV standard was assayed in parallel to participant samples as a positive control [14]. Results were analysed using QIAcuity Software Suite (Version 2.1.7).

### Quantification of HIV DNA in human brain tissue

Droplet digital PCR (QX200; BioRad, Hercules, CA) was used for the quantification of HIV DNA and RPP30 DNA in human brain tissue, as previously described [8]. Intact, 3’ defective and 5’ defective HIV proviral DNA in human brain tissue was quantified by IPDA, as previously described [8].

### Detection of HIV p24 proteins in human brain tissue

Immunofluorescence was performed on 5 μm serial sections of FFPE human brain tissue mounted on Superfrost Plus microscope slides (Fisher Scientific). Slides were dewaxed in xylene for 6 minutes then rehydrated in 1 minute washes of descending EtOH concentrations (100%, 90%, 70%, 50%), then rinsed in H_2_O. Slides were heated to 95°C for 20 minutes in antigen retrieval buffer (pH6) and cooled to room temperature. Tissues were incubated in 3% H_2_O_2_ for 10 minutes, then washed in 0.1% TBS-tween20. The primary antibody (p24; 1:10; cat: M0857; DAKO) was added to each slide and incubated for 2 hours. The slides were washed in 0.1% TBS-tween20 for 5 minutes. Primary antibody was detected with biotinylated goat anti-rabbit/mouse IgG (cat: ab64257; Abcam) and HRP labelled streptavidin (cat: ab64269; Abcam). This two-step process included 2x 10 minute incubations, with a 5 minute 0.1% TBS-tween20 wash between steps. Opal fluorophore (1,200; cat: FP1488001KT; Akoya) was used to visualize p24. To remove residual antibody for the next round of antigen detection, each slide was boiled for 20 minutes in antigen retrieval buffer and left to cool at room temperature. This method was repeated for the second primary antibody (CD68; 1:200; 2 hours, cat: M0814; DAKO) and the third primary antibody (CD3; 1:100; 2 hours; cat: A0452; DAKO). Nuclei was counterstained with Hoechst 33258 (1,750; 10 minutes; cat: H3569; Invitrogen) and lipofuscin was quenched with True Black (1,20 in 70% ethanol; 30 seconds; cat: 23007; Biotium). Slides were rinsed in H_2_O and mounted with Fluoromount G (cat: 495802; Invitrogen). Mounted slides were dried overnight and scanned at 40x magnification (PALM; Zeiss).

### Statistics

Comparisons were made using non-parametric Mann-Whitney (unpaired) or Wilcoxon tests (paired) using GraphPad Prism analysis software (version 9.2.0, GraphPad Software, La Jolla California USA). P<0.05 considered statistically significant. Linear regression analysis or Spearman correlation were performed to determine the association between variables.

## Supporting information

S1 Fig HIV RNA copies measured by RNA input and per million cells measured by DNA mass. HIV RNA transcripts for HIV TAR, Long-LTR, Pol, PolyA and Tat/Rev quantified from frontal cortex brain tissue in **(A and B)** non-virally suppressed (n = 13) or (**C and D**) virally suppressed PWH (n = 10). HIV transcripts were standardised by RNA input and by 10^6^ cell equivalents as measured by DNA mass. **(E and F)** HIV RNA transcripts (as above) quantified in matched PBMCs from virally suppressed PWH and standardised by RNA input or by 10^6^ cell equivalents as measured by DNA mass. Comparisons are using non-parametric paired Wilcoxon-tests where coloured symbols represent individual PWH. Median and interquartile ranges shown. *P<0.05, **P<0.01, ***P<0.001.
(TIF)

S2 Fig Levels of HIV RNA transcripts in brain tissue are directly associated with intact HIV DNA levels in PWH. Linear regression analysis of HIV RNA transcription **(A)** initiation (TAR), **(B)** elongation (Long-LTR), **(C)** Pol, **(D)** completion (PolyA) and **(E)** multiply spliced Tat/Rev transcripts vs intact HIV DNA levels in brain tissue as measured by digital PCR for virally suppressed (n = 2–7; blue symbols) and non-virally suppressed PWH (n = 6–9; red symbols). All parameters log transformed; R^2^, P values and 95% confidence intervals shown.
(TIF)

S3 Fig PBMCs exhibit a higher ratio of HIV RNA TAR/total DNA in PBMCs than matched frontal cortex brain tissue from virally suppressed PWH. **(A)** Ratio of HIV RNA HIV TAR, Long-LTR, Pol, PolyA and Tat/Rev transcripts relative to total HIV DNA quantified by QIAcuity digital PCR from frontal cortex tissue (n = 10) or **(B)** PBMCs from virally suppressed PWH (n = 5). HIV transcripts standardised to 10^6^ cell equivalents as measured by RRP30 DNA. Colored symbols represent individual PWH. **(C)** Comparative analysis of the ratio of HIV RNA TAR, Long-LTR, Pol, PolyA and multiply spliced Tat/Rev HIV transcripts relative to total HIV DNA between frontal cortex brain tissue (green) and PBMCs (orange). Comparisons made by Mann-Whitney U tests. Median and interquartile ranges shown. *P<0.05; **P<0.01.
(TIF)

S1 Table Correlative analysis between HIV RNA transcripts in the brain and clinical parameters. (DOCX)
