# Supplementary figures and images for: HIV transcription persists in the brain of virally suppressed people with HIV
Source: PLoS Pathog. 2024 Aug 8;20(8):e1012446. doi: 10.1371/journal.ppat.1012446 (PMC11335163; doi:10.1371/journal.ppat.1012446)

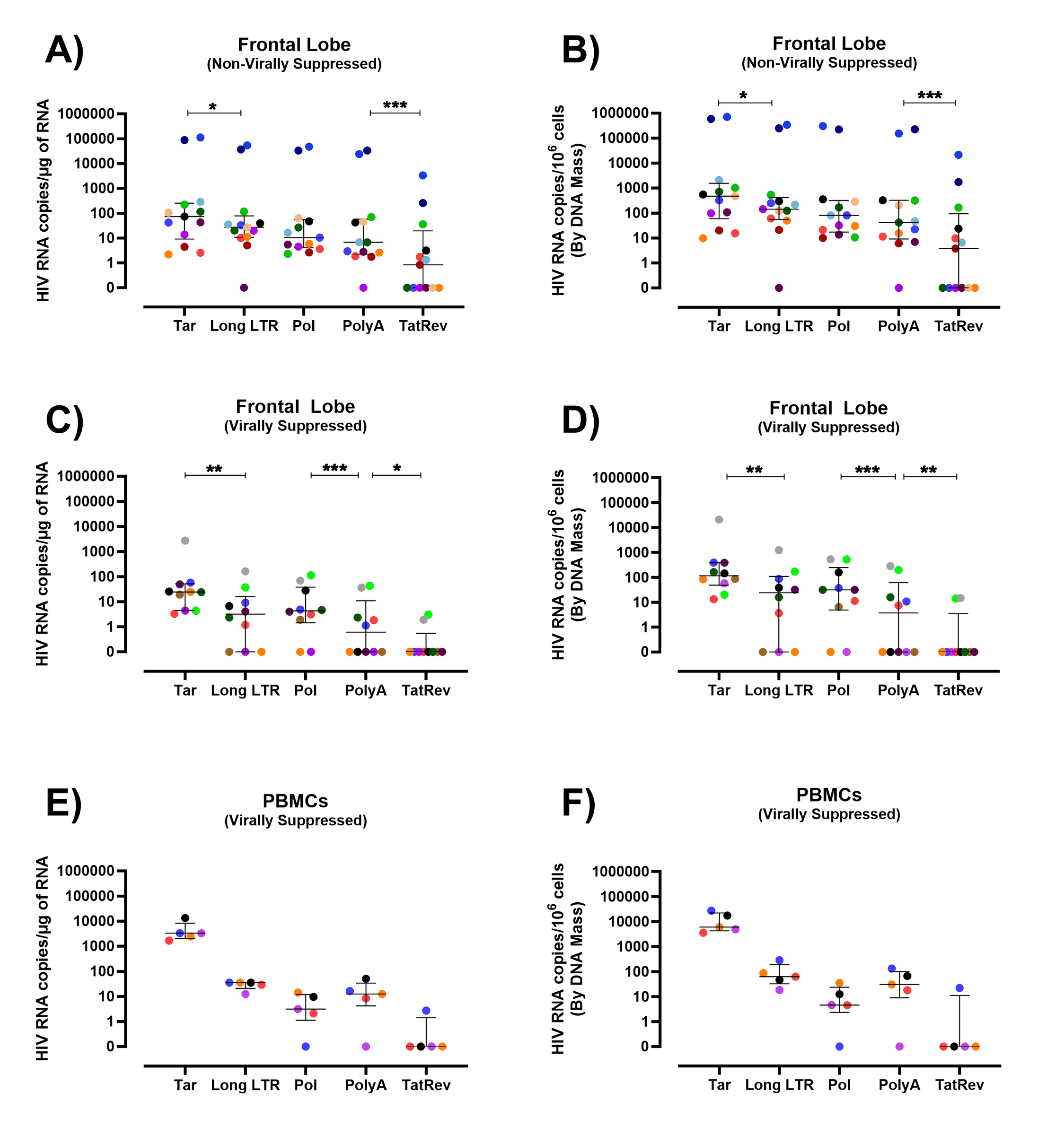

Supplement: S1 Fig — HIV RNA transcripts for HIV TAR, Long-LTR, Pol, PolyA and Tat/Rev quantified from frontal cortex brain tissue in (A and B) non-virally suppressed (n = 13) or (C and D) virally suppressed PWH (n = 10). HIV transcripts were standardised by RNA input and by 106 cell equivalents as measured by DNA mass. (E and F) HIV RNA transcripts (as above) quantified in matched PBMCs from virally suppressed PWH and standardised by RNA input or by 106 cell equivalents as measured by DNA mass. Comparisons are using non-parametric paired Wilcoxon-tests where coloured symbols represent individual PWH. Median and interquartile ranges shown. *P<0.05, **P<0.01, ***P<0.001. (TIF) [file ppat.1012446.s001.tif]

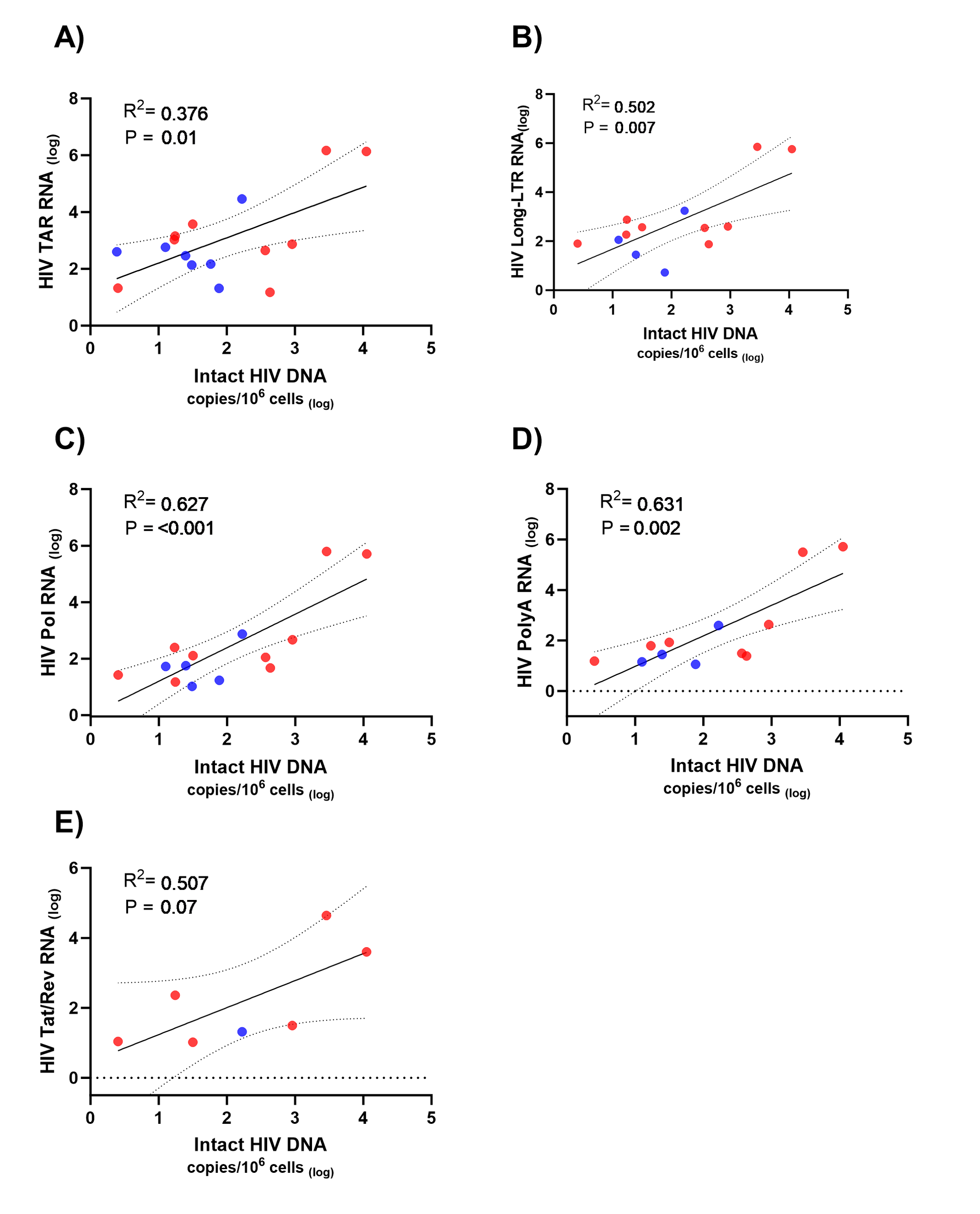

Supplement: S2 Fig — Linear regression analysis of HIV RNA transcription (A) initiation (TAR), (B) elongation (Long-LTR), (C) Pol, (D) completion (PolyA) and (E) multiply spliced Tat/Rev transcripts vs intact HIV DNA levels in brain tissue as measured by digital PCR for virally suppressed (n = 2–7; blue symbols) and non-virally suppressed PWH (n = 6–9; red symbols). All parameters log transformed; R2, P values and 95% confidence intervals shown. (TIF) [file ppat.1012446.s002.tif]

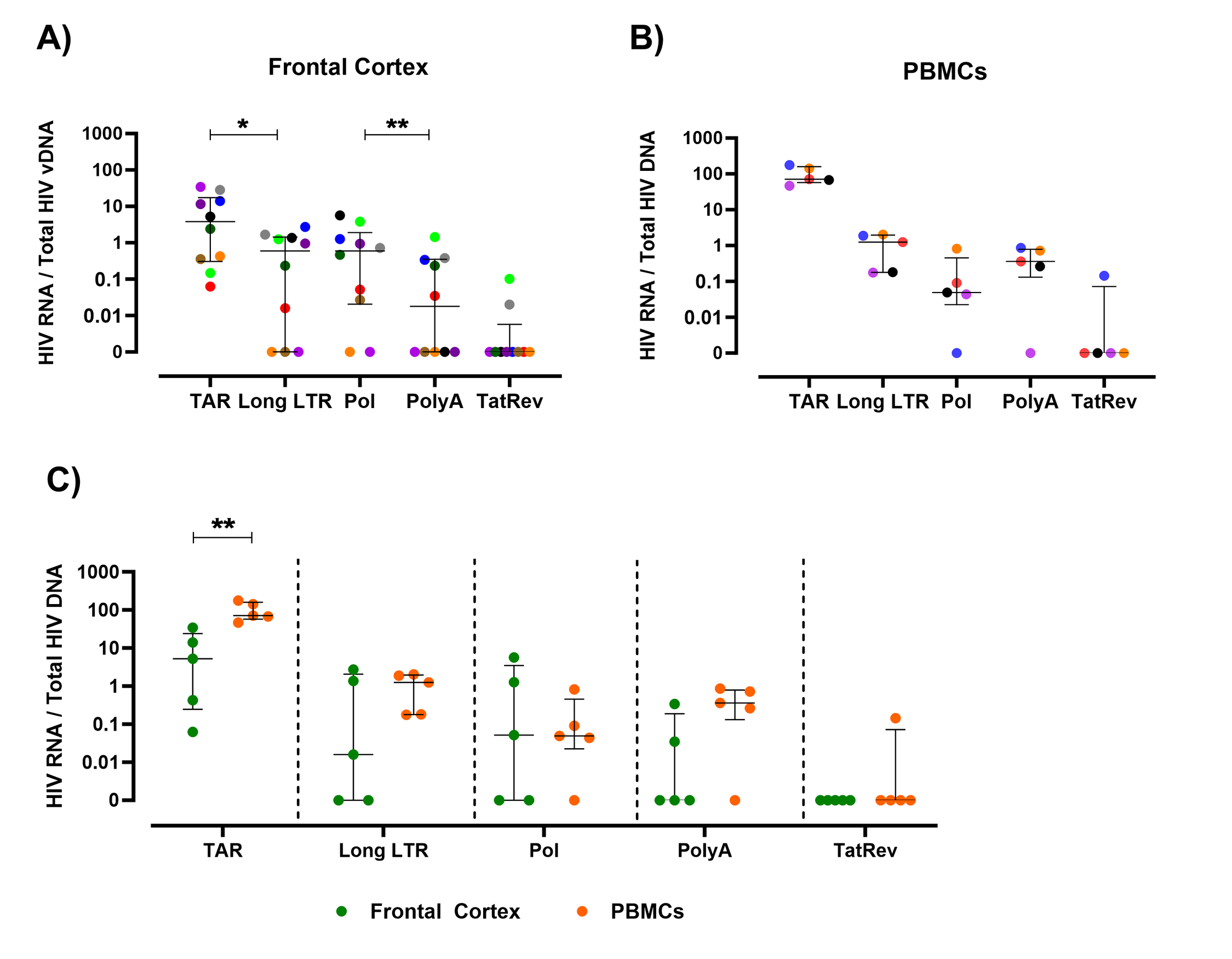

Supplement: S3 Fig — (A) Ratio of HIV RNA HIV TAR, Long-LTR, Pol, PolyA and Tat/Rev transcripts relative to total HIV DNA quantified by QIAcuity digital PCR from frontal cortex tissue (n = 10) or (B) PBMCs from virally suppressed PWH (n = 5). HIV transcripts standardised to 106 cell equivalents as measured by RRP30 DNA. Colored symbols represent individual PWH. (C) Comparative analysis of the ratio of HIV RNA TAR, Long-LTR, Pol, PolyA and multiply spliced Tat/Rev HIV transcripts relative to total HIV DNA between frontal cortex brain tissue (green) and PBMCs (orange). Comparisons made by Mann-Whitney U tests. Median and interquartile ranges shown. *P<0.05; **P<0.01. (TIF) [file ppat.1012446.s003.tif]
