# Supplementary material for: HIV transcription persists in the brain of virally suppressed people with HIV
Source: PLoS Pathog. 2024 Aug 8;20(8):e1012446. doi: 10.1371/journal.ppat.1012446 (PMC11335163; doi:10.1371/journal.ppat.1012446)
Supplement: S1 Table — (DOCX) [file ppat.1012446.s004.docx]

| **Table S1. Correlative analysis between HIV RNA transcripts in the brain and clinical parameters** | | | | | | | | | | | | | | | | | | | |
| --- | --- | --- | --- | --- | --- | --- | --- | --- | --- | --- | --- | --- | --- | --- | --- | --- | --- | --- | --- |
|  | **TAR** | | |  | **Long-LTR** | | |  | **Pol** | | |  | **PolyA** | | |  | **Tat-Rev** | | |
| **Parameter** | **N** | ***ρ*** | **P** |  | **N** | ***ρ*** | **P** |  | **N** | ***ρ*** | **P** |  | **N** | ***ρ*** | **P** |  | **N** | ***ρ*** | **P** |
| Age | 23 | -0.345 | 0.106 |  | 19 | -0.314 | 0.190 |  | 21 | -0.094 | 0.684 |  | 16 | -0.205 | 0.444 |  | 9 | 0.368 | 0.327 |
| CD4+ T cells _(/mm_^3^_)_ | 23 | 0.004 | 0.986 |  | 19 | -0.278 | 0.249 |  | 21 | -0.269 | 0.238 |  | 16 | -0.152 | 0.573 |  | 9 | -0.008 | 0.992 |
| Nadir CD4+ T cells _(/mm_^3^_)_ | 13 | -0.220 | 0.470 |  | 10 | -0.503 | 0.144 |  | 11 | -0.527 | 0.100 |  | 9 | -0.383 | 0.313 |  | 5 | -0.600 | 0.35 |
| Plasma viral load ^a^  ^(HIV RNA copies/mL)^ | 13 | 0.017 | 0.964 |  | 12 | 0.211 | 0.510 |  | 13 | 0.359 | 0.228 |  | 11 | 0.220 | 0.516 |  | 7 | 0.523 | 0.240 |
| Plasma viral load _(days)_ | 23 | -0.046 | 0.836 |  | 19 | 0.058 | 0.814 |  | 21 | 0.230 | 0.315 |  | 16 | 0.024 | 0.935 |  | 9 | 0.183 | 0.644 |
| CPE score^b^ | 18 | 0.046 | 0.856 |  | 14 | 0.146 | 0.617 |  | 16 | 0.123 | 0.647 |  | 11 | 0.205 | 0.543 |  | 6 | -0.493 | 0.333 |

LTR: long terminal repeat; PolyA: polyadenylation; TAR: transactivation response element

Spearman rho and P value shown. Virally suppressed and non-virally suppressed individuals combined for analysis

^a^ Non-virally suppressed PWH only

^b^ Virally suppressed PWH only
